# Kidney disease screening at ART initiation among adults with HIV in Uganda: A missed priority for a high-risk population

**DOI:** 10.1371/journal.pgph.0005106

**Published:** 2026-02-02

**Authors:** Grace Kansiime, Joseph Baruch Baluku, Edwin Nuwagira, Michael Kanyesigye, Paul Stephen Obwoya, Rose Muhindo, Winnie R. Muyindike, Pliers Denis Tusingwire, Henry Mugerwa, Matthew Odera, Monday Busuulwa, Matthew Ssemakadde, Esther C. Atukunda, Francis Bajunirwe, Robert Kalyesubula, Mark J. Siedner

**Affiliations:** 1 Department of Medicine, Mbarara University of Science and Technology, Mbarara, Uganda; 2 Division of Pulmonology, Kiruddu National Referral Hospital, Kampala, Uganda; 3 Global Health Collaborative, Mbarara University of Science and Technology, Mbarara, Uganda; 4 Department of Medicine, Mbarara Regional Referral Hospital, Mbarara, Uganda; 5 Department of Ophthalmology, Kabale University School of Medicine, Kabale, Uganda; 6 Joint Clinical Research Centre, Kampala, Uganda; 7 AIDS Healthcare Foundation Uganda Cares, Masaka Regional Referral Hospital, Masaka, Uganda; 8 Department of Pharmacy, Mbarara University of Science and Technology, Mbarara, Uganda; 9 Department of Community Health, Mbarara University of Science and Technology, Mbarara, Uganda; 10 Department of Physiology and Department of Medicine, Makerere University College of Health Sciences, Kampala, Uganda; 11 Division of Infectious Diseases, Department of Medicine, Massachusetts General Hospital, Boston, Massachusetts, United States of America; 12 Harvard Medical School, Boston, Massachusetts, United States of America; Weill Cornell Medicine, UNITED STATES OF AMERICA

## Abstract

Kidney disease affects 850 million people worldwide, with Sub-Saharan Africa bearing a significant burden. People living with HIV (PWH) are at increased risk due to nephrotoxicity of antiretroviral therapy (ART), in part due to widespread use of tenofovir disoproxil fumarate. In response, Uganda recommends routine kidney disease screening by doing a serum creatinine test at ART initiation. However, the extent of adherence to these guidelines remains poorly understood. We extracted clinical data for adults initiating ART between 2017 and 2024 at three large-volume HIV clinics in Uganda. To determine if kidney disease screening rates had increased appropriately over time, we divided the observation period into three eras as per national guidelines: (1) Test and Treat (2017–2019), that recommended screening only PWH and diabetes or hypertension; (2) DTG rollout/COVID-19 (2020–2022); and (3) creatinine-for-all (2023–2024), recommending screening everyone initiating ART. Logistic regression models were fit to identify correlates of renal screening. Of the 17,485 participants, only 22.4% (3,909/17,485) were screened for kidney disease at ART initiation. Screening was more common at the urban site (54.2%) compared to rural sites (10.0%). At rural sites, screening declined over time and individuals were 83% less likely to be screened in the creatinine-for-all era compared to the baseline era (aOR 0.17, 95% CI: 0.13–0.22) while it increased at urban site (aOR 9.27, 95% CI: 7.37–11.66). Male sex (aOR 1.37, 95% CI: 1.20–1.57), older age (≥45 years), hypertension, and non–TDF-based ART regimens were associated with higher screening odds at rural sites. Diabetes, opportunistic infections, and TDF use were not significantly associated with screening likelihood at any site. Kidney disease screening for PWH at ART initiation remains poor in Uganda, even when using a single creatinine test, particularly in rural clinics, highlighting critical challenges in translating national guidelines into practice. Future research should focus on understanding multilevel barriers to screening and evaluating strategies to improve guideline uptake.

## Background

Kidney disease is a growing challenge globally, affecting 850 million people worldwide and contributing significantly to morbidity and mortality [1–4]. Sub-Saharan Africa (SSA) bears a disproportionately high burden of chronic kidney disease (CKD), with prevalence estimates exceeding 15% [5–7]. The increased rates of CKD in SSA are attributed to rising prevalence of diabetes and hypertension, regionally common genetic predispositions such as sickle cell trait and APOL 1, and endemic infections such as tuberculosis, malaria, schistosomiasis, hepatitis B and C, and HIV [7–12].

SSA bears the highest global burden of HIV. Over half of the people living with HIV (PWH) worldwide reside in eastern and southern Africa [13,14]. PWH are at increased risk for CKD due to direct pathogenicity of the HIV, coinfections such as hepatitis B and C, comorbidities such as diabetes and hypertension as well as nephrotoxicity of antiretroviral therapy (ART) agents, notably tenofovir disoproxil fumarate (TDF), the main backbone of ART in SSA [15–18]. Late diagnosis and referral for nephrology care among PWH may worsen the prognosis of CKD, leading to complications of cardiovascular disease, bone disorders, dyslipidemia, cognitive decline, and decreased quality of life [19–23].

In response, many countries, including Uganda, recommend routine kidney disease screening before starting ART (at ART initiation) as well as regular monitoring while on ART. In Uganda, guidelines recommend doing a creatinine test for all PWH at ART initiation and yearly while on ART for kidney disease screening purposes [24]. However, whether such guidelines are followed in people initiating ART is not well studied. We hypothesized that kidney disease screening at ART initiation remains low for PWH in Uganda, despite the advancement of guidelines to do so. To test this hypothesis, we evaluated routine kidney disease screening among PWH in Uganda over the past eight years (2017–2024) in three large-volume HIV clinics in Uganda.

## Methods

### Study setting and participants

We conducted data analysis for a retrospective cohort of PWH on ART at three large volume HIV clinics in Uganda: Mbarara Regional Referral Immune Suppression (ISS) clinic, Masaka Regional Referral Hospital (RRH) HIV clinic as rural clinics, and Joint Clinical Research Centre (JCRC) HIV clinic an urban clinic, with combined patient census of 50,000. We included all adults aged 18 years and above, initiated on ART between January 2017 and December 2024.

### Data collection methods

All three clinics have electronic medical records systems that record patients’ clinical encounters, medications prescribed, and results of laboratory tests. We extracted demographic and clinical data for all adult patients who started ART during the observation period. Extracted indicators included; ART initiation date, creatinine test dates and results, age at ART initiation, sex, education level, marital status, occupation, weight, height, alcohol use and smoking history, co-morbidity history (hypertension, diabetes, tuberculosis, cryptococcal meningitis, and Kaposi sarcoma), CD4 count at ART initiation, and baseline ART regimen.

### Statistical methods

Data were analyzed using Stata software, version SE 17.1. We first summarized continuous variables using medians and interquartile ranges (IQR), and categorical variables using percentages. We subsequently examined changes in kidney disease screening at ART initiation over time in Uganda. Our primary outcome of interest was completion of kidney disease screening, which we defined as having a serum creatinine test done (as per Uganda guidelines) within three months of ART initiation. Our primary exposure of interest was the ART initiation period, which we defined a priori as three era:. 1) the “test and treat” era, where all PWH were eligible for ART regardless of CD4 count and creatinine screening was recommended for those with comorbid diabetes or hypertension; 2) the “DTG and COVID-19”, era where all individuals were started on TLD, but health systems were constrained due to the COVID-19 pandemic; and 3) the “creatinine screening for all” era when creatinine screening for all individuals starting ART was first implemented (Table 1).

**Table 1:** Study time periods of interest.

Years	2017–2019	2020–2022	2023–2024
Study Period	**Test and Treat era**	**DTG and Covid-19 era**	**Creatinine for all era**
Uganda ART Guidelines and other era considerations	All PWH eligible for therapy. First-line regimen: efavirenz, tenofovir, and lamivudine	All PWH eligible for therapy. First-line regimen: dolutegravir, tenofovir, and lamivudine. Some care interruptions due to COVID-19 lockdowns	All PWH eligible for therapy. First-line regimen: dolutegravir, tenofovir, and lamivudine.
Uganda kidney disease screening guidelines for PWH	Serum creatinine for PWH with comorbid diabetes or hypertension [25].	Serum creatinine for PWH with comorbid diabetes or hypertension [26].	Serum creatinine screening for all PWH at time of ART initiation [24].

ART, Antiretroviral therapy; DTG, dolutegravir; PWH, people with HIV.

Crude rates of kidney disease screening were estimated by clinic type and period. Multivariable logistic regression models were utilized to estimate the association between selected covariates and kidney disease screening at ART initiation. Notably, a significant qualitative interaction was observed between creatinine screening changes over time and the urban (JCRC) versus rural (Mbarara and Masaka) sites. Consequently, these were treated as distinct populations.

### Human subjects and ethical considerations

The study was reviewed and approved by the Mbarara University Review and Ethics Committee (MUST ID: 2024–1667) and received a waiver of informed consent. Additionally, we obtained approval from the Uganda National Council of Science and Technology (HS5305ES), and administrative clearances from all the study sites.

## Results

### Study population

Between January and May 2025, we extracted data for 17,485 adults who were initiated on ART from January 2017 to December 2024 at Mbarara RRH, Masaka RRH, and JCRC HIV clinics in Uganda. The baseline and clinical characteristics of participants were largely similar across the three ART initiation periods (Table 2). Most participants (n = 11,068, 63%) were female, and the majority (n = 14,907, 85.2%) were younger than 45 years of age. The median CD4 count was 378 (interquartile range [IQR] 172–631) cells/uL, and less than 10% (n = 1,319) had diabetes or hypertension. For those with available sociodemographic data, about half (6,349/11,572, 54.9%) had a primary level education or less. Most (10,841/12,052, 90.0%) reported being employed.

**Table 2:** Participant baseline characteristics.

	Study period			
	Test & treat era n=8,164	DTG/COVID-19 era n=5,764	Creatinine for all era n=3,557	Total N = 17,485
**Sex**				
Male	2,921 (35.8%)	2,118 (36.7%)	1,378 (38.7%)	6,417 (36.7%)
Female	5,243 (64.2%)	3,646 (63.3%)	2,179 (61.3%)	11,068 (63.3%)
**Age at ART start, median (IQR)**	30 (25–38)	32 (26–40)	32 (25.3-40)	31 (25–39)
**Age category (years**				
18-24	2,040 (25.0%)	1,186 (20.6%)	766 (21.5%)	3,992 (22.8%)
25-44	5,103 (62.5%)	3,594 (62.4%)	2,218 (62.4%)	10,915 (62.4%)
≥ 45	1,021 (12.5%)	984 (17.1%)	573 (16.1%)	2,578 (14.7%)
**Education n = 11,572**				
Primary and below	3,567 (56.9%)	1,955 (51.8%)	827 (53.9%)	6,349 (54.9%)
Secondary	2,162 (34.5%)	1,395 (37.0%)	495 (32.2%)	4,052 (35.0%)
Tertiary	537 (8.6%)	421 (11.2%)	213 (13.9%)	1,171 (10.1%)
**Study site**				
ISS Mbarara	3,009 (36.9%)	1,792 (31.1%)	1,001 (28.1%)	5,802 (33.2%)
Masaka	3,609 (44.2%)	1,998 (34.7%)	1,069 (30.1%)	6,676 (38.2%)
JCRC	1,546 (18.9%)	1,974 (34.2%)	1,487 (41.8%)	5,007 (28.6%)
**Occupation, n = 12,052**				
Unemployed	690 (11.1%)	364 (9.8%)	157 (7.4%)	1,211 (10.0%)
Business	1,897 (30.5%)	1,169 (31.4%)	736 (34.9%)	3,802 (31.5%)
Farmer	1,318 (21.2%)	683 (18.4%)	309 (14.6%)	2,310 (19.2%)
Other	2,317 (37.2%)	1,504 (40.4%)	908 (43.0%)	4,729 (39.2%)
**Marital status n = 8,494**				
Not married	1,831 (47.9%)	1,463 (50.6%)	980 (55.0%)	4,274 (50.3%)
Married	1,991 (52.1%)	1,428 (49.4%)	801 (45.0%)	4,220 (49.7%)
**Baseline CD4 (cell/**µ**L) median (IQR)**	389 (181-666)	378 (175-624)	359 (148-597)	378 (172-631)
**CD4 category n = 10,166**				
<200	1,050 (26.8%)	1,011 (28.4%)	812 (30.2%)	2,873 (28.3%)
200-500	1,334 (34.0%)	1,271 (35.7%)	958 (35.6%)	3,563 (35.0%)
>500	1,535 (39.2%)	1,277 (35.9%)	918 (34.2%)	3,730 (36.7%)
**Comorbidities**				
Hypertension	459 (5.6%)	288 (5.0%)	164 (4.6%)	911 (5.2%)
Diabetes mellitus	171 (2.1%)	155 (2.7%)	82 (2.3%)	408 (2.3%)
Tuberculosis	635 (7.8%)	533 (9.2%)	343 (9.6%)	1,511 (8.6%)
Cryptococcal meningitis	145 (1.8%)	128 (2.2%)	31 (0.9%)	304 (1.7%)
Kaposi sarcoma	92 (1.1%)	48 (0.8%)	10 (0.3%)	150 (0.9%)
**Baseline Regimen n = 16,212**				
TDF based	7,423 (97.9%)	5,180 (98.3%)	3,317 (98.7%)	15,920 (98.2%)
Non-TDF based	158 (2.1%)	91 (1.7%)	43 (1.3%)	292 (1.8%)

ART, Antiretroviral therapy; CD4, Cluster of differentiation; DM, diabetes mellitus; DTG, dolutegravir; HTN, hypertension; ISS, Immune suppression syndrome, IQR, interquartile range, JCRC, Joint clinical research centre; PWH, people with HIV; TDF, tenofovir disoproxil fumurate; TLD, Tenofovir Lamivudine Dolutegravir; TLE, Tenofovir Lamivudine, Efavirenz; yrs, years.

### Prevalence of kidney disease screening in Uganda

**Fig 1:**
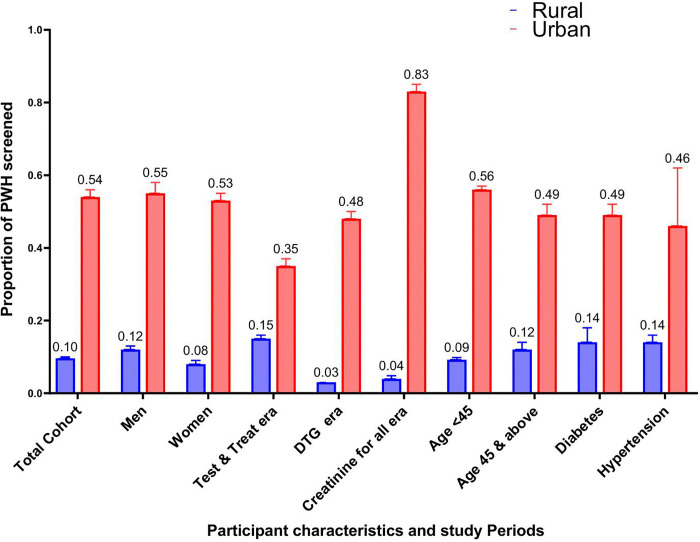
Proportion of PWH screened for kidney disease at ART initiation at rural and urban sites.

The proportion of the total population who completed kidney disease screening within three months of ART initiation during the observation period was 22.4% (3,909/17,485, 95%CI 21.7 – 23.0). At the urban site, 54.2% (95%CI 52.8-55.6) completed kidney disease screening compared to 9.6% (95%CI 9.1-10.1) at the rural sites (Fig 1).

**Fig 2:**
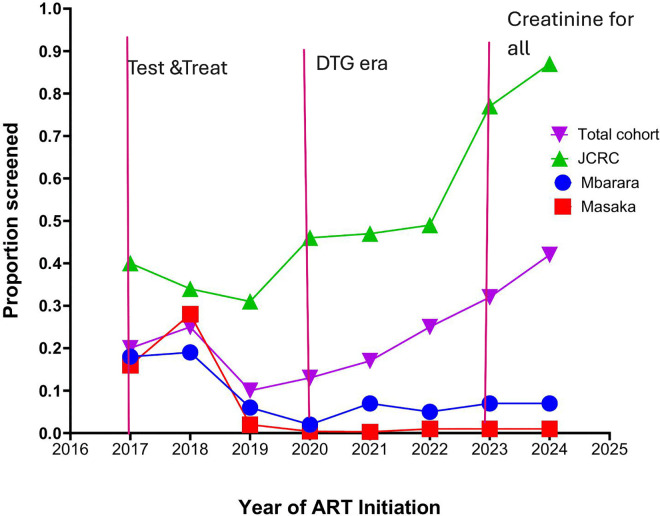
Kidney disease screening trends for PWH in Uganda by ART initiation year.

Similarly, the crude occurrence of kidney disease screening was consistently higher over the study observation period at the urban site, while it declined or remained stagnant at the rural sites (Fig 2).

### Factors associated with kidney disease screening in Uganda

At rural study sites, kidney disease screening rates declined over time. Compared to the initial test and treat period, participants initiating ART in the creatinine-for-all era had an 83% lower likelihood of kidney disease screening (aOR 0.17, 95% CI: 0.13 - 0.22, p < 0.001, Table 3). In contrast, the urban site showed a substantial increase in screening during the creatinine-for-all period, with participants over nine times more likely to be screened than in the test and treat era (aOR 9.27, 95% CI: 7.37-11.66, p < 0.001, Table 4).

**Table 3:** Correlates of kidney screening at rural Uganda clinics.

Baseline characteristics	Proportion screened% (n/N)	Univariable Models			Multivariable Models		
Total Cohort	9.6 (1,196/12,478)	OR	95%CI	p-value	AOR	95%CI	p-value
**Sex**							
Female	8.4 (681/8,126)	Ref					
Male	11.8 (515/4, 352)	1.467	1.300 - 1.656	<0.001	1.371	1.202 - 1.566	<0.001
**Age category, yrs**							
18-24	7.7 (250/3,251)	Ref					
25-44	10.1 (846/8,409)	1.343	1.159 - 1.556	<0.001	1.191	1.016 - 1.396	0.031
45 and above	12.2 (100/818)	1.672	1.308 - 2.138	<0.001	1.393	1.061 - 1.828	0.017
**Study site**							
ISS Mbarara	10.4 (602/5,802)	Ref					
Masaka	8.9 (594/6,676)	0.844	0.749 - 0.951	0.005	0.920	0.810 - 1.045	0.200
**Baseline CD4 (cell/µL)**							
<200	10.8 (251/2,330)	Ref					
200-500	10.7 (306/2,861)	0.992	0.831 - 1.184	0.929	1.034	0.858 - 1.246	0.727
>500	11.4 (270/2,379)	1.060	0.884 - 1.272	0.528	1.070	0.879 - 1.302	0.500
Missing	7.5 (369/ 4,908)	0.673	0.569 - 0.797	<0.001	0.494	0.413 - 0.592	<0.001
**Comorbidities**							
**HTN**							
No	10.1 (1,075/11,602)	Ref					
Yes	13.8 (121/876)	1.569	1.283 - 1.921	<0.001	1.327	1.066 - 1.652	0.011
**DM**							
No	9.5 (1,155/12,183)	Ref					
Yes	14.3 (40/280)	1.592	1.133 - 2.237	0.007	1.437	0.994 - 2.079	0.054
**TB**							
No	9.6 (1,060/ 11,002)	Ref					
Yes	9.2 (136/1,476)	0.952	0.789 - 1.148	0.606			
**CCM**							
No	9.6 (1,162/12,174)	Ref					
Yes	11.2 (34/304)	1.193	0.831 - 1.714	0.338			
**KS**							
No	9.6 (1,182/12,343)	Ref					
Yes	10.4 (14/135)	1.093	0.626 - 1.906	0.755			
**ART Regimen n = 12,425**							
TDF based	9.5 (1,161/12,241)	Ref					
Non-TDF based	16.2 (30/185)	1.859	1.252 - 2.762	0.002	1.660	1.091 - 2.526	0.018
**Study period**							
Test & treat era	15.4 (1,020/6,618)	Ref					
DTG era	2.5 (95/3,790)	0.141	0.114 - 0.175	<0.001	0.123	0.099 - 0.153	<0.001
Creatinine for all	3.9 (81/2,070)	0.224	0.177 - 0.282	<0.001	0.170	0.134 - 0.215	<0.001

**Table 4:** Correlates of kidney screening at a large urban Ugandan clinic.

Baseline characteristics	Proportion screened; % (n/N)	Univariable Models			Multivariable Models		
Total Cohort	54.2 (2,713/5,007)	OR	95%CI	p-value	AOR	95%CI	p-value
**Sex**							
Female	53.4 (1,570/2,942)	Ref					
Male	55.4 (1,143/2,065)	1.083	0.968 - 1.213	0.165	1.195	1.023 - 1.397	0.025
**Age category, yrs**							
18-24	56.8 (421/741)	Ref					
24-44	55.4 (1,981/3,573)	0.946	0.806 - 1.110	0.494	0.888	0.709 - 1.112	0.300
45 and above	44.9 (311/693)	0.620	0.502 - 0.762	<0.001	0.567	0.425 - 0.757	<0.001
**Baseline CD4 (cell/µL)**							
<200	76.8 (417/543)	Ref					
200-500	77.1 (541/702)	1.015	0.778 - 1.325	0.911	1.018	0.734 - 1.410	0.916
>500	64.9 (877/1,351)	0.559	0.445 - 0.703	<0.001	0.685	0.518 – 0.906	0.008
Missing	36.4 (878/ 2,411)	0.173	0.140 - 0.215	<0.001	0.255	0.196 - 0.333	<0.001
**Comorbidities**							
**HTN**							
No	54.2 (2,697/4,972)	Ref					
Yes	45.7 (16/35)	0.710	0.364 - 1.385	0.315	1.497	0.553 - 4.056	0.427
**DM**							
No	54.3 (2,650/4,879)	Ref					
Yes	49.2 (63/128)	0.815	0.574 - 1.158	0.254	0.767	0.461 - 1.278	0.309
**TB**							
No	54.3 (2,701/ 4,972)	Ref					
Yes	34.3 (12/35)	0.439	0.218 - 0.884	0.021	0.414	0.190 - 0.906	0.027
**KS**							
No	54.3 (2,708/4,992)	Ref					
Yes	33.3 (5/15)	0.422	0.144 - 1.236	0.115			
**ART Regimen**							
TDF based	70.5 (2,592/3,679)	Ref					
Non-TDF based	51.4 (55/107)	0.444	0.302 - 0.652	<0.001	0.793	0.522 - 1.206	0.279
Missing	5.14 (66/1,221)	0.240	0.019 -0.031	<0.001	0.026	0.020 - 0.034	<0.001
**Study period**							
“Test & treat” era	34.6 (535/1,546)	Ref					
DTG era	47.9 (946/1,974)	1.739	1.516 - 1.994	<0.001	1.209	1.022 - 1.430	0.027
“Creatinine for all” era	82.9 (1,232/1,487)	9.130	7.697 - 10.830	<0.001	9.272	7.372 - 11.662	<0.001

ART, Antiretroviral therapy; AOR, adjusted odds ratio; CI, confidence interval; CD4, Cluster of differentiation; DM, diabetes mellitus; DTG, dolutegravir; HTN, hypertension; ISS, Immune suppression syndrome; IQR, interquartile range; JCRC, Joint clinical research centre; KS, Kaposi sarcoma; OR, odds ratio; PWH, people with HIV; TB, tuberculosis; TDF, tenofovir disoproxil fumarate; yrs, years.

Several factors were associated with higher odds of kidney disease screening at ART initiation in rural sites (Table 3). For example, male participants were about 40% more likely to be screened than females (aOR 1.37, 95% CI: 1.20–1.57, p < 0.001), as were those aged 45 years and above compared to those below 25 years (aOR 1.39, 95% CI: 1.06–1.83, p = 0.017). Individuals with hypertension also had a 33% increased likelihood of screening (aOR 1.33, 95% CI: 1.07–1.65, p = 0.011), and those initiated on non–TDF-based regimens were significantly more likely to be screened (aOR 1.66, 95% CI: 1.09–2.53, p = 0.018). In contrast, having diabetes or HIV-associated opportunistic infections did not affect one’s odds of getting kidney disease screening.

At the urban site, participants with higher CD4 counts (>500 cells/µL) were significantly less likely to be screened compared to those with CD4 < 200 cells/µL (aOR 0.69, 95% CI: 0.52–0.91, p = 0.008, Table 4). However, having hypertension, diabetes, and opportunistic infections, or initiating a TDF-based ART regimen was not associated with increased odds of kidney disease screening.

## Discussion

In this analysis of data from three large HIV clinics caring for approximately 50,000 PWH in Uganda, we found a low overall prevalence of kidney disease screening at ART initiation. Screening was particularly rare at rural sites where screening rates were less than 10%, and have remained low since 2022, when guidelines officially recommended kidney disease screening for all. Despite the presence of guidelines and low-cost, evidence-based interventions to slow CKD progression, these data would suggest that the health system in Uganda is missing crucial opportunities to improve primary care management for PWH, such as kidney disease screening, identification of CKD and opportunities to improve longterm care of those with the condition.[27].

Our findings support those of other studies, indicating that most patients with kidney disease are not screened and are diagnosed with advanced kidney disease, when treatment options are limited [28–30]. Such low rates of screening are not unique to PWH, but also extend to other high-risk groups in the region [27,31,32]. Indeed, only a few countries, such as South Africa and Egypt, have established national screening programs for high-risk populations, and achieved adequate CKD screening rates [33,34]. Cameroon recently rolled out a free national screening program as well [35,36]. However, most countries, including Uganda, Kenya, and Ghana, report sporadic kidney disease screening programs for high risk populations typically championed by local kidney disease associations during awareness campaigns [37–40]. The low rates of screening and early detection of reversible kidney disease can be particularly devastating in low- and middle income countries (LMICs), where fragile healthcare systems are already strained, exposing individuals and families to catastrophic treatment costs and poor outcomes associated with advanced kidney disease [27,41–45].

There is limited data regarding kidney disease screening for PWH from high-income countries (HICs). A study from Australia reports kidney disease screening rates above 90% [46]. Most kidney disease screening studies from HICs have been among other high-risk populations, such as those with diabetes mellitus and hypertension [47–49]. The reported high rates of screening in these studies highlight the established kidney disease screening programs in HICs. The decline and/or stagnation in CKD screening rates at rural health facilities in recent years, despite updated guidelines and increased advocacy, is particularly concerning. While the coronavirus-2019 (COVID-19) pandemic may have strained health systems there should be visible trends of recovery post the pandemic, which were not observed in this study at rural sites, which serve the majority of PWH in Uganda.

Our analysis of rural Ugandan health facilities reveals that kidney disease screening disproportionately targets patients with traditional risk factors, such as male gender, older age (45 + years), or comorbid hypertension. This suggests that, in resource-limited settings, healthcare providers may be prioritizing screening for individuals perceived to be at highest clinical risk, possibly reflecting older guidelines that targeted screening based on comorbidities rather than current universal recommendations. Intriguingly, individuals initiated on a non-TDF ART regimen were 1.7 times more likely to be screened, perhaps reflecting provider suspicion of underlying renal dysfunction that influenced baseline creatinine testing and regimen selection. Conversely, the large urban health facility in our study demonstrated dramatically improved rates of screening than the rural centers, highlighting how local capacity can shape guideline adherence. At this facility, screening rates increased substantially in the “creatinine for all” era to approximately 80% in the most recent years, reflecting a successful scale-up and implementation of the updated national guidelines. Yet, even here, screening remained selective. Participants with higher CD4 counts (>500 cells/µL) were 30% less likely to be screened than those with advanced HIV (CD4 < 200 cells/µL), and those aged ≥45 or with tuberculosis were significantly under-screened. These patterns, especially the lower screening rate among older adults and those with TB suggest clinical triage based on perceived acuity, despite guidelines and known renal risks [24,50,51]. Even in well-resourced settings, provider discretion and patient characteristics continue to shape screening decisions more than guidelines alone. These screening trends may also reflect gaps in knowledge and awareness of guideline updates, underscoring the need for emphasis on unique risk factors such as HIV and ART in high-burden settings [28,52–56] Systemic health sector constraints, such as unreliable laboratory supply chains and stock-outs in rural areas, may also be contributing.

An important area for future research will be to explore the factors that facilitate and restrict kidney disease screening in the rural health facilities of HIV care in the region. This information may help inform strategies to increase routine screening in practice. In addition, clinics could implement quality improvement methods to track and report kidney disease screening as well as implementing electronic health records’ alerts if creatinine is deranged. This information may help clinicians to identify PWH and kidney disease to facilitate early intervention.

Our study has several limitations. Firstly, our definition of kidney disease screening relied solely on serum creatinine testing (as this was the only measure captured in the electronic medical records), without urine albumin/protein or tubular function markers. Secondly, because the data were derived from clinical databases, they may not fully reflect or capture all cases of kidney disease screening. For instance, due to the absence of a national health insurance scheme and amidst frequent stock-outs of diagnostic supplies, some participants may have undergone screening tests at private facilities, which may not be captured in routine clinical data systems. Additionally, data clerks manually enter results after they are printed as hard copies, potentially leading to an underestimation of true kidney disease screening rates. These limitations mean that true kidney disease screening rates are likely lower than reported here. Thirdly, we were unable to obtain data on key contextual variables that may have contributed to the observed low screening rates, such as the availability of diagnostic resources at each facility or the composition and training levels of healthcare providers involved in ART initiation during the study period. As a result, we cannot report on the influence of these factors on screening practices. Nevertheless, we present data from three large public-sector HIV clinics in Uganda, spanning urban and rural settings and reflecting routine clinical practice and use of programmatic data that includes a broad patient population, enhancing the generalizability of our findings to comparable settings.

In summary, we found a low overall prevalence of kidney disease screening for PWH at ART initiation in Uganda, particularly at rural sites where screening rates remain below 10%. These findings provide insights for health system strengthening for kidney disease in the region, where care delivery and adherence to clinical guidelines are often influenced by factors such as donor support, diagnostic resource availability, and local infrastructure constraints. Future research should investigate the underlying reasons for low and selective kidney disease screening, especially in rural areas. Implementation science approaches may help identify health system, provider, and patient-level barriers influencing screening uptake. Prospective studies using both serum creatinine and urine albumin creatinine ratio are also needed to better quantify CKD burden in HIV populations in Uganda and the region, to ensure the optimization of the health and well-being of this high-risk population. We recommend that the Ugandan government considers adopting pragmatic interventions such as integrating **urine dipstick testing** alongside **serum creatinine measurement** at ART initiation in line with global standards, and scaling up **point-of-care creatinine testing** in all HIV care settings, especially rural facilities, to enable early detection and management of kidney disease

## Supporting information

S1 Table Participant baseline characteristics stratified by rural vs urban location. (DOCX)

S1 Data Dataset used in manuscript preparation. (CSV)

## References

[1] Jager KJ, Kovesdy C, Langham R, Rosenberg M, Jha V, Zoccali C. A single number for advocacy and communication—worldwide more than 850 million individuals have kidney diseases. Oxford University Press. 2019. 10.1093/ndt/gfz174 31566230

[2] Bello AK, Okpechi IG, Levin A, Ye F, Damster S, Arruebo S, et al. An update on the global disparities in kidney disease burden and care across world countries and regions. Lancet Glob Health. 2024;12(3):e382–95. doi: 10.1016/S2214-109X(23)00570-3 38365413

[3] Cockwell P, Fisher L-A. The global burden of chronic kidney disease. Lancet. 2020;395(10225):662–4. doi: 10.1016/S0140-6736(19)32977-0 32061314

[4] Kidney disease: a global health priority. Nat Rev Nephrol. 2024;20(7):421–3. doi: 10.1038/s41581-024-00829-x 38570630

[5] Kaze AD, Ilori T, Jaar BG, Echouffo-Tcheugui JB. Burden of chronic kidney disease on the African continent: a systematic review and meta-analysis. BMC Nephrol. 2018;19(1):125. doi: 10.1186/s12882-018-0930-5 29859046 PMC5984759

[6] Matsha TE, Erasmus RT. Chronic kidney disease in sub-Saharan Africa. Lancet Glob Health. 2019;7(12):e1587–8. doi: 10.1016/S2214-109X(19)30467-X 31708128

[7] Xie Y, Bowe B, Mokdad AH, Xian H, Yan Y, Li T, et al. Analysis of the Global Burden of Disease study highlights the global, regional, and national trends of chronic kidney disease epidemiology from 1990 to 2016. Kidney international. 2018;94(3):567–81. 30078514 10.1016/j.kint.2018.04.011

[8] Network GC. Global Burden of Disease Study 2019 (GBD 2019) Burden by Risk 1990–2019. Seattle, WA: Institute for Health Metrics and Evaluation (IHME). 2020.

[9] Arogundade FA, Hassan MO, Omotoso BA, Oguntola SO, Okunola OO, Sanusi AA, et al. Spectrum of kidney diseases in Africa: malaria, schistosomiasis, sickle cell disease, and toxins. Clin Nephrol. 2016;86 (2016)(13):53–60. doi: 10.5414/CNP86S120 27509585

[10] Ulasi II, Awobusuyi O, Nayak S, Ramachandran R, Musso CG, Depine SA. Chronic kidney disease burden in low-resource settings: regional perspectives. Elsevier. 2022. 10.1016/j.semnephrol.2023.151336 37058859

[11] George JA, Brandenburg J-T, Fabian J, Crowther NJ, Agongo G, Alberts M, et al. Kidney damage and associated risk factors in rural and urban sub-Saharan Africa (AWI-Gen): a cross-sectional population study. Lancet Glob Health. 2019;7(12):e1632–43. doi: 10.1016/S2214-109X(19)30443-7 31708144 PMC7033368

[12] Jagannathan R, Patzer RE. Urbanization and kidney function decline in low and middle income countries. BMC Nephrol. 2017;18(1):276. doi: 10.1186/s12882-017-0685-4 28851306 PMC5576323

[13] World Health Organisation. HIV and AIDS key facts. July 2024.

[14] UNAIDS. AIDS AT A CROSSROADS: UNAIDS Global AIDS Update 2024. (accessed May 10, 2025). 2024.Available online at: https://www.unaids.org/en/resources/documents/2024/global-aids-update-2024

[15] Wu P-Y, Hung C-C, Liu W-C, Hsieh C-Y, Sun H-Y, Lu C-L, et al. Metabolic syndrome among HIV-infected Taiwanese patients in the era of highly active antiretroviral therapy: prevalence and associated factors. J Antimicrob Chemother. 2012;67(4):1001–9. doi: 10.1093/jac/dkr558 22232517

[16] Mtisi TJ, Ndhlovu CE, Maponga CC, Morse GD. Tenofovir-associated kidney disease in Africans: a systematic review. AIDS Res Ther. 2019;16(1):12. doi: 10.1186/s12981-019-0227-1 31171021 PMC6554992

[17] Eshetu B, Gedefie A, Mulatie Z, Alemayehu E, Belete MA, Tekele SG, et al. Prevalence of chronic kidney diseases among people living with HIV in Ethiopia: a systematic review and meta-analysis. BMC Infect Dis. 2025;25(1):850. doi: 10.1186/s12879-025-11212-x 40597745 PMC12219290

[18] Abate MD, Kassa MA, Yilak G, Habtie TE, Temesgen D, Mulu B, et al. Incidence, progression and predictors of chronic kidney disease among adult HIV/AIDS patients on antiretroviral treatment in comprehensive specialised hospitals in the Amhara Region, Ethiopia, 2022: a multi-centre retrospective follow-up study. BMJ Open. 2025;15(7):e090345. doi: 10.1136/bmjopen-2024-090345 40633949 PMC12278170

[19] McGettrick P, Alvarez Barco E, Mallon PW, editors. Ageing with HIV. Healthcare; 2018: MDPI. 10.3390/healthcare6010017 PMC5872224 29443936

[20] Tannor EK, Norman BR, Adusei KK, Sarfo FS, Davids MR, Bedu-Addo G. Quality of life among patients with moderate to advanced chronic kidney disease in Ghana - a single centre study. BMC Nephrol. 2019;20(1):122. doi: 10.1186/s12882-019-1316-z 30961570 PMC6454740

[21] Bagasha P, Leng M, Katabira E, Petrova M. Health-related quality of life, palliative care needs and 12-month survival among patients with end stage renal disease in Uganda: protocol for a mixed methods longitudinal study. BMC Nephrol. 2020;21(1):531. doi: 10.1186/s12882-020-02197-7 33287725 PMC7720495

[22] Ripley E. Complementary effects of angiotensin-converting enzyme inhibitors and angiotensin receptor blockers in slowing the progression of chronic kidney disease. Am Heart J. 2009;157(6 Suppl):S7–16. doi: 10.1016/j.ahj.2009.04.008 19450722

[23] Yamagata K, Iseki K, Nitta K, Imai H, Iino Y, Matsuo S, et al. Chronic kidney disease perspectives in Japan and the importance of urinalysis screening. Clin Exp Nephrol. 2008;12(1):1–8. doi: 10.1007/s10157-007-0010-9 18175065

[24] MOH. Consolidated guidelines for the prevention and treatment of HIV and AIDS in Uganda. Ministry Of Health, Uganda.; 2022.

[25] MOH. Consolidated guidelines for the prevention and treatment of HIV and AIDS in Uganda. Kampala: Ministry Of Health, Uganda. 2016.

[26] MOH. Consolidated guidelines for the prevention and treatment of HIV and AIDS in Uganda. Ministry of Health Uganda. 2020.

[27] Bello A, Okpechi I, Levin A, Ye F, Saad S, Zaidi D. ISN–Global Kidney Health Atlas: a report by the International Society of Nephrology: an assessment of global kidney health care status focussing on capacity, availability, accessibility, affordability and outcomes of kidney disease. Brussels: International Society of Nephrology. 2023.

[28] Babua C, Kalyesubula R, Okello E, Kakande B, Sebatta E, Mungoma M, et al. Pattern and presentation of cardiac diseases among patients with chronic kidney disease attending a national referral hospital in Uganda: a cross sectional study. BMC Nephrol. 2015;16:126. doi: 10.1186/s12882-015-0128-z 26238594 PMC4522958

[29] Marie Patrice H, Joiven N, Hermine F, Jean Yves B, Folefack François K, Enow Gloria A. Factors associated with late presentation of patients with chronic kidney disease in nephrology consultation in Cameroon-a descriptive cross-sectional study. Ren Fail. 2019;41(1):384–92. doi: 10.1080/0886022X.2019.1595644 31106687 PMC6534206

[30] Halle MP, Essomba N, Djantio H, Tsele G, Fouda H, Luma NH, et al. Clinical characteristics and outcome of HIV infected patients with chronic kidney disease in Sub Saharan Africa: an example from in Cameroon. BMC Nephrol. 2019;20(1):253. doi: 10.1186/s12882-019-1446-3 31288761 PMC6617860

[31] López DS, Vargas JAH, Urina-Jassir M, Urina-Triana M, Franco OH. Reducing the gap of chronic kidney disease in low-and middle-income countries: what is missing?. The Lancet Regional Health–Americas. 2023;28. 10.1016/j.lana.2023.100625 PMC10638012 37969876

[32] Maritim PK, Twahir A, Davids MR. Global Dialysis Perspective: Kenya. Kidney360. 2022;3(11):1944–7. 36514403 10.34067/KID.0006662021 PMC9717619

[33] Brennan AT, Kileel EM, Khoza S, Crowther NJ, Bor J, Fox MP, et al. Prevalence and progression of chronic kidney disease among adults undergoing creatinine testing in South African public healthcare facilities: a study leveraging data from South Africa’s National Health Laboratory Service (NHLS). BMJ Public Health. 2024;2(1):e000799. doi: 10.1136/bmjph-2023-000799 39698394 PMC11654635

[34] Soliman AR, Soliman KM, Abdelaziz TS, Ahmed RM, Abdellatif DA, Darwish RA, et al. The evolution of nephrology practice in Egypt: legacy, current challenges, and future directions-a narrative review. Ren Fail. 2025;47(1):2509784. doi: 10.1080/0886022X.2025.2509784 40438027 PMC12123898

[35] Princeley. N. August 25, 2025.Princeley N. 2025.

[36] Mbunwe T. Kidney disease screening project targets 30,000, in SW. Littoral. 2025.

[37] Kabinga SK, McLigeyo SO, Twahir A, Ndungu JN, Wangombe NN, Nyarera DK, et al. Risk factors for chronic kidney disease in the community: A decade of outreach in Kenya. Clinical Epidemiology and Global Health. 2024;30:101823. doi: 10.1016/j.cegh.2024.101823

[38] Batte A, Gyagenda JO, Otwombe K, Muhindo R, Bagasha P, Kiggundu D, et al. Prevalence and predictors of hypertension among adults in Mbarara City, Western Uganda. Chronic Illn. 2023;19(1):132–45. doi: 10.1177/17423953211058408 34786975

[39] Tannor EK, Calice-Silva V. Kidney Health for All-Efforts in Low-Income Settings to Enhance Community Engagement, Kidney Health Awareness, and Screening. Kidney Int Rep. 2021;7(3):359–62. doi: 10.1016/j.ekir.2021.12.017 35257047 PMC8897301

[40] Otieno FCF, Ogola EN, Kimando MW, Mutai K. The burden of unrecognised chronic kidney disease in patients with type 2 diabetes at a county hospital clinic in Kenya: implications to care and need for screening. BMC Nephrol. 2020;21(1):73. doi: 10.1186/s12882-020-1705-3 32111192 PMC7048110

[41] Kalyesubula R, Brewster U, Kansiime G. Global dialysis perspective: Uganda. Kidney360. 2022;3(5):933–6. 36128482 10.34067/KID.0007002021 PMC9438417

[42] Jardine T, Davids MR. Global dialysis perspective: South Africa. Kidney360. 2020;1(12). 10.34067/KID.0005152020 PMC8815536 35372888

[43] Okoro RN. Cushioning the economic burden of chronic kidney disease for patients in LMICs: The heightened need for a government-driven financial support policy. Health Policy and Technology. 2021;10(2):100507. doi: 10.1016/j.hlpt.2021.100507

[44] Kalyesubula R, Aklilu AM, Calice-Silva V, Kumar V, Kansiime G. The Future of Kidney Care in Low- and Middle-Income Countries: Challenges, Triumphs, and Opportunities. Kidney360. 2024;5(7):1047–61. doi: 10.34067/KID.0000000000000489 38922683 PMC11296549

[45] Agada-Amade YA, Ogbuabor DC, Eboreime E, Onwujekwe OE. Cost analysis of the management of end-stage renal disease patients in Abuja, Nigeria. Cost Eff Resour Alloc. 2023;21(1):94. doi: 10.1186/s12962-023-00502-3 38066603 PMC10704650

[46] Gracey D, Chan D, Bailey M, Richards D, Dalton B. Screening and management of renal disease in human immunodeficiency virus-infected patients in Australia. Intern Med J. 2013;43(4):410–6. doi: 10.1111/j.1445-5994.2012.02933.x 22931386

[47] Edmonston D, Lydon E, Mulder H, Chiswell K, Lampron Z, Marsolo K, et al. Concordance With Screening and Treatment Guidelines for Chronic Kidney Disease in Type 2 Diabetes. JAMA Netw Open. 2024;7(6):e2418808. doi: 10.1001/jamanetworkopen.2024.18808 38922613 PMC11208975

[48] Folkerts K, Petruski-Ivleva N, Comerford E, Blankenburg M, Evers T, Gay A, et al. Adherence to Chronic Kidney Disease Screening Guidelines Among Patients With Type 2 Diabetes in a US Administrative Claims Database. Mayo Clin Proc. 2021;96(4):975–86. doi: 10.1016/j.mayocp.2020.07.037 33722396

[49] Peralta CA, Frigaard M, Rolon L, Seal K, Tuot D, Senyak J, et al. Screening for CKD To Improve Processes of Care among Nondiabetic Veterans with Hypertension: A Pragmatic Cluster-Randomized Trial. Clin J Am Soc Nephrol. 2020;15(2):174–81. doi: 10.2215/CJN.05050419 32034070 PMC7015085

[50] MOH. Integrated chronic kidney disease management guidelines for Uganda. Ministry of Health, Uganda. June; 2024.

[51] Kansiime G, Aklilu AM, Baluku JB, Yasmin F, Kanyesigye M, Muzoora CK, et al. Incidence of Acute Kidney Injury and Associated Mortality among Individuals with Drug-Susceptible Tuberculosis in Uganda. Kidney360. 2024;5(10):1446–54. doi: 10.34067/KID.0000000000000551 39141693 PMC11556924

[52] Komenda P, Ferguson TW, Macdonald K, Rigatto C, Koolage C, Sood MM, et al. Cost-effectiveness of primary screening for CKD: a systematic review. Am J Kidney Dis. 2014;63(5):789–97. doi: 10.1053/j.ajkd.2013.12.012 24529536

[53] Levin A, Stevens PE, Bilous RW, Coresh J, De Francisco AL, De Jong PE, et al. KDIGO 2012 clinical practice guideline for the evaluation and management of chronic kidney disease. Kidney International Supplements. 2013;3(1):1–150.

[54] Shlipak MG, Tummalapalli SL, Boulware LE, Grams ME, Ix JH, Jha V, et al. The case for early identification and intervention of chronic kidney disease: conclusions from a Kidney Disease: Improving Global Outcomes (KDIGO) Controversies Conference. Kidney Int. 2021;99(1):34–47. doi: 10.1016/j.kint.2020.10.012 33127436

[55] Kidney Disease: Improving Global Outcomes (KDIGO) CKD Work Group. KDIGO 2024 Clinical Practice Guideline for the Evaluation and Management of Chronic Kidney Disease. Kidney Int. 2024;105(4S):S117–314. doi: 10.1016/j.kint.2023.10.018 38490803

[56] Ramkelawan V, Mbeje PN, Mtshali NG. Recommendation to improve chronic kidney disease management guideline in primary healthcare, KwaZulu-Natal. Curationis. 2025;48(1):e1–8. doi: 10.4102/curationis.v48i1.2623 40035110 PMC11886468

