# Supplementary material for: Kidney disease screening at ART initiation among adults with HIV in Uganda: A missed priority for a high-risk population
Source: PLOS Glob Public Health. 2026 Feb 2;6(2):e0005106. doi: 10.1371/journal.pgph.0005106 (PMC12863478; doi:10.1371/journal.pgph.0005106)
Supplement: S1 Table — (DOCX) [file pgph.0005106.s001.docx]

**S1 Table:** Participant baseline characteristics stratified by rural vs urban location

| **Baseline characteristics** | **Study site** | | **Total**  N=17,485 |
| --- | --- | --- | --- |
|  | **Rural**  (Mbarara, Masaka)  n=12,478 | **Urban**  (JCRC) n=5,007 |  |
| **Sex** |  |  |  |
| Male | 4,352 (34.9%) | 2,065 (41.2%) | 6,417 (36.7%) |
| Female | 8,126 (65.1%) | 2,942 (58.8%) | 11,068 (63.3%) |
| **Age at ART start, median (IQR); yrs** | 30 (24-37) | 34.9 (28.1-43.7) | 31 (25-39) |
| **Age category** |  |  |  |
| 18-24 | 3,251 (26.1%) | 741 (14.8%) | 3,992 (22.8%) |
| 25-44 | 8,409 (67.4%) | 3,573 (71.4%) | 11,982 (68.5%) |
| 45 and above | 818 (6.6%) | 693 (13.8%) | 1,511 (8.6%) |
| **Education n=11,572** |  |  |  |
| Primary and below | 5,772 (56.3%) | 577 (43.8%) | 6,349 (54.9%) |
| Secondary | 3,527 (34.4%) | 525 (39.9%) | 4,052 (35.0%) |
| Tertiary | 957 (9.3%) | 214 (16.3%) | 1,171 (10.1%) |
| **Occupation, n=12,052** |  |  |  |
| Unemployed | 1,211 (11.3%) | 0 (0.0%) | 1,211 (10.0%) |
| Business | 2,958 (27.5%) | 844 (65.3%) | 3,802 (31.5%) |
| Farmer | 2,211 (20.6%) | 99 (7.7%) | 2,310 (19.2%) |
| Other | 4,379 (40.7%) | 350 (27.1%) | 4,729 (39.2%) |
| **Marital status n= 8,494** |  |  |  |
| Not married | 2,841 (49.3%) | 1,433 (52.5%) | 4,274 (50.3%) |
| Married | 2,925 (50.7%) | 1,295 (47.5%) | 4,220 (49.7%) |
| **Baseline CD4 (cell/µL); median (IQR)** | 341 (150-565) | 522 (248-950) | 378 (172-631) |
| **CD4 category** |  |  |  |
| <200 | 2,330 (18.7%) | 543 (10.8%) | 2,873 (16.4%) |
| 200-500 | 2,861 (22.9%) | 702 (14.0%) | 3,563 (20.4%) |
| >500 | 2,379 (19.1%) | 1,351 (27.0%) | 3,730 (21.3%) |
| Missing | 4,908 (39.3%) | 2,411 (48.2%) | 7,319 (41.9%) |
| **Comorbidities** |  |  |  |
| Hypertension | 876 (7.0%) | 35 (0.7%) | 911 (5.2%) |
| Diabetes mellitus | 280 (2.2%) | 128 (2.6%) | 408 (2.3%) |
| Tuberculosis | 1,476 (11.8%) | 35 (0.7%) | 1,511 (8.6%) |
| Cryptococcal meningitis | 304 (2.4%) | 0 (0%) | 304 (1.7%) |
| Kaposi sarcoma | 135 (1.1%) | 15 (0.3%) | 150 (0.9%) |
| **Study Period** |  |  |  |
| Test and treat era | 6,618 (53.0%) | 1,546 (30.9%) | 8,164 (46.7%) |
| DTG era | 3,790 (30.4%) | 1,974 (39.4%) | 5,764 (33.0%) |
| Creatinine for all era | 2,070 (16.6%) | 1,487 (29.7%) | 3,557 (20.3%) |
| **Baseline Regimen n= 16,212** |  |  |  |
| TDF based | 12,242 (98.5%) | 3,679 (97.2%) | 15,921 (98.2%) |
| Non-TDF based | 184 (1.5%) | 107 (2.8%) | 291 (1.8%) |

*ART, Antiretroviral therapy; CD4, Cluster of differentiation; DM, diabetes mellitus; DTG, dolutegravir; HTN, hypertension; ISS, Immune suppression syndrome; IQR, interquartile range; JCRC, Joint clinical research centre; KS, Kaposi sarcoma; PWH, people with HIV; TB, tuberculosis; TDF, tenofovir disoproxil fumarate; yrs, years*
